# Feeding mice with diets containing mercury-contaminated fish flesh from French Guiana: a model for the mercurial intoxication of the Wayana Amerindians

**DOI:** 10.1186/1476-069X-7-53

**Published:** 2008-10-29

**Authors:** Jean-Paul Bourdineaud, Nadège Bellance, Giovani Bénard, Daniel Brèthes, Masatake Fujimura, Patrice Gonzalez, Aline Marighetto, Régine Maury-Brachet, Cécile Mormède, Vanessa Pédron, Jean-Nicolas Philippin, Rodrigue Rossignol, William Rostène, Masumi Sawada, Muriel Laclau

**Affiliations:** 1 Université de Bordeaux 1-CNRS UMR 5805, Station Marine d'Arcachon, place du Docteur Peyneau, Arcachon, 33120, France; 2 Physiopathologie Mitochondriale, Université Victor Segalen Bordeaux2-INSERM U688, 146 rue Léo Saignat, Bordeaux, 33076 cedex, France; 3 Institut de Biochimie et Génétique Cellulaires, Université Victor Segalen Bordeaux 2, 1 rue Camille Saint-Saëns, Bordeaux, 33077 cedex, France; 4 National Institute for Minamata Disease, Pathology Section, Department of Basic Medical Sciences, 4058-18 Hama, Minamata, Kumamoto 867-0008, Japan; 5 Laboratoire de Neurosciences Cognitives, Université de Bordeaux 1-CNRS UMR 5106, Avenue des Facultés, Talence, 33405, France; 6 Centre de Recherches Saint-Antoine, INSERM U732, Hôpital Saint-Antoine, 184 rue du Faubourg Saint-Antoine, Paris, 75571 cedex 12, France

## Abstract

**Background:**

In 2005, 84% of Wayana Amerindians living in the upper marshes of the Maroni River in French Guiana presented a hair mercury concentration exceeding the limit set up by the World Health Organization (10 μg/g). To determine whether this mercurial contamination was harmful, mice have been fed diets prepared by incorporation of mercury-polluted fish from French Guiana.

**Methods:**

Four diets containing 0, 0.1, 1, and 7.5% fish flesh, representing 0, 5, 62, and 520 ng methylmercury per g, respectively, were given to four groups of mice for a month. The lowest fish regimen led to a mercurial contamination pressure of 1 ng mercury per day per g of body weight, which is precisely that affecting the Wayana Amerindians.

**Results:**

The expression of several genes was modified with mercury intoxication in liver, kidneys, and hippocampus, even at the lowest tested fish regimen. A net genetic response could be observed for mercury concentrations accumulated within tissues as weak as 0.15 ppm in the liver, 1.4 ppm in the kidneys, and 0.4 ppm in the hippocampus. This last value is in the range of the mercury concentrations found in the brains of chronically exposed patients in the Minamata region or in brains from heavy fish consumers. Mitochondrial respiratory rates showed a 35–40% decrease in respiration for the three contaminated mice groups. In the muscles of mice fed the lightest fish-containing diet, cytochrome *c *oxidase activity was decreased to 45% of that of the control muscles. When mice behavior was assessed in a cross maze, those fed the lowest and mid-level fish-containing diets developed higher anxiety state behaviors compared to mice fed with control diet.

**Conclusion:**

We conclude that a vegetarian diet containing as little as 0.1% of mercury-contaminated fish is able to trigger in mice, after only one month of exposure, disorders presenting all the hallmarks of mercurial contamination.

## Background

Methylmercury is a neurotoxic compound, which has been shown to be the cause of the Minamata disease. Diseased persons were struck by ataxia and suffered from visual, sensorial and hearing problems, seizures, memory disabilities, muscular weakness and cramps [1]. The effects of low amplitude prenatal exposure on neurological development have been described, and human exposure to methylmercury has been linked to fish and shellfish consumption [2].

In French Guiana, clandestine gold mining contaminates numerous sites, both terrestrial and aquatic. Divalent and organic mercury can then enter and pollute biological systems. In last instances, Amerindian populations are contaminated after consumption of carnivorous fish. The mercurial contamination of 35 fish species caught in the Courcibo River, free of gold mining, and Leblond River, whose banks are the location of intensive gold mining, in French Guiana was analyzed and a relationship was found with the level of each species among the trophic web. Results showed a mercury amplification all along the food web: the ratio between the extreme muscle mercury concentrations in piscivorous species (14.3 μg/g dry weight, for *Acestrorhynchus guianensis*) and herbivorous species (0.02 μg/g dw, for *Myleus ternetzi*) was 715 [3]. The final predators in this food web are human beings, and consequently high mercury levels are always quantified in hair of Amerindian community members [4]. In 1997, 64% of Wayana Amerindians living in the upper marshes of the Maroni River presented a hair mercury concentration exceeding the safety limit set up by the World Health Organization, and above which adverse effects on brain development are likely to occur (10 μg/g or 10 ppm) [5]. In 2005, this proportion reached 84%, indicating that the problem of mercury contamination increases with time. All individuals one year and older were ingesting, through fish consumption, a mercury dose more important than the security limit set to 200 μg/week. Four carnivorous fish species, *Pseudoplatystoma fasciatum*, *Hoplias aimara*, *Ageneiosus brevifilis*, and *Serrasalmus rhombeus*, represented at least 72% of the total mercury ingested by the Wayana families [6]. A survey has been carried out in French Guiana showing a significant correlation between mercury contamination levels and neurological impairments. Amerindian children from the upper Maroni were highly contaminated with a mean of 12 ppm in hair, and were afflicted by neurological disorders such as poorer coordination of the legs, and decreased performance in the Stanford-Binet copying score [7]. Taking this correlation into consideration, our long-term goal is to ascertain whether the mercury found in the fish consumed by the Wayana Amerindians is the source of the observed troubles, and if so whether this mercurial intoxication observed among the Amerindian populations is likely to endanger their lives.

To achieve this objective, we chose the rodent model (mouse). The idea was to mimic as closely as possible the Wayanas' contamination mode. Therefore, we decided to incorporate lyophilized fish flesh into the preparation of mice alimentary pellets. This flesh originated from fish contaminated by mercury in their natural habitat and caught in the Sinnamary River in French Guiana. More precisely, the *Hoplias aimara *species, which Amerindians are fond of, was chosen because this fish is highly contaminated by methylmercury (4 to 12 μg/g dw), and because this single species represents 27% of the Wayanas' dietary mercury intake and 10.7% of the total flesh they consume [6]. A more classical approach consisting in dispersing a given quantity of methylmercury within diet preparations was precluded because the supramolecular form under which methylmercury enters the body is of crucial importance. Indeed it has been shown that methylmercury contained in fish flesh was mainly associated to proteinaceous aliphatic thiols [8]. Therefore, one can suspect a different trophic transfer rate through the intestinal barrier, and a different early toxicity for ingested free and protein-bound methylmercury. In line with this, a higher faecal excretion and lower tissue accumulation, as well as metallothionein induction in rats following exposure to methylmercury naturally incorporated in fish compared to methylmercury chloride added to the same matrix have been reported [9].

Dietary MeHg is readily and efficiently absorbed by the human gastrointestinal tract, to a reported level of 95% to 100% [10]. However, nothing is known about the MeHg trophic transfer rate in mice at such low exposure doses. Thus, to establish our model, and although our long-term objective was a follow-up of the dietary MeHg impact on mice metabolism all along the animals' life, we first had to determine the fish regimen that given to mice would as closely as possible mimic a human contamination. More precisely, the main goal of this experiment was to select the MeHg-contaminated diet leading to kidney and brain Hg concentrations in the range of what has been recorded in human kidneys and brains of heavy fish consumers in a general population [11,12].

Three fish flesh-containing diets were made up from a basic vegetarian diet. These diets incorporated 0.1, 1, and 7.5% lyophilized *H. aimara *flesh, yielding mercury concentrations of 5.4, 62, and 520 ng per g of food pellets, respectively. After feeding mice one month with such regimens, the effects of mercury-containing fish flesh as compared to the control diet were assessed through tissue mercury content analysis, gene expression quantification, muscle mitochondrial respiration assays, and tests for anxiety.

## Methods

### Preparation of the mice diets

In French Guiana, a survey of the daily mercury intake in the Wayana Amerindian population has been carried out. Adult men aged between 25 to 45 years were daily ingesting a mean of 61 μg mercury [6]. Their mean body weight being around 60 kg, the mercurial contamination pressure was 1 ng Hg/day/g body weight. For mice weighing around 25 g, such a dose corresponds to a daily ingestion of 25 ng mercury brought by a mean consumption of 5 g pellets. Therefore, to mimic the Wayanas' contamination, mice food pellets had to contain 5 ng Hg/g brought by dry fish flesh supplementation. The *H. aimara *fish whose flesh was used was caught in French Guiana in the Sinnamary River, known to be contaminated by methylmercury mostly originating from the Petit-Saut hydroelectric reservoir [13]. The dry flesh of this animal contained 5 μg Hg/g. Thus, a diet containing 0.1% of this fish flesh could mimic Wayana's contamination. However, the main goal of our study was to first select a Hg diet content resulting in mice brain and kidneys mercury accumulations of the same amplitude as that measured in human heavy fish consumers. We thus chose to have prepared three diets supplemented with 0.1, 1, and 7.5% of lyophilized fish flesh, along with a control diet devoid of flesh. These special diets have been manufactured by Special Diets Services (Witham, Essex, United Kingdom; French commercial representation: Dietex, Saint-Gratien, France). The control diet was mainly vegetarian (Rat and Mouse n°1 maintenance diet, abbreviated to RM1 diet, Special Diets Services). According to Special Diets Services, RM1 diet is made by blending wheat, barley, wheatfeed, de-hulled extracted toasted soya, soya protein concentrate, macro and micro minerals, soya oil, whey powder, amino acids, and vitamins. The nutrient compositions of the control RM1 and the three prepared regimens are given in Table 1 (the analyses were carried out by Special Diets Services). A macro analysis of lyophilized *H. aimara *flesh nutrients has also been done. This flesh contains: 8.1% moisture, 1.8% crude fat, and 89.9% crude protein. A comparison of the diets' compositions showed that there were no substantial differences between the control and the low and mid level diets. The main difference between the control and the high level diets lies in the crude protein content: 14% versus 20%, respectively. We quantified the total mercury content of the three prepared regimens, and found 5.4 ± 0.5, 62.4 ± 12.8, and 520 ± 187 ng Hg/g of food pellets for the diets containing 0.1, 1, and 7.5% fish flesh, respectively. The control RM1 diet contained 1.4 ± 0.2 ng Hg/g of food pellets. The mercury species contained in the control RM1 diet is the inorganic form since it is the one accumulated by plants whereas the contribution of the methylated species to the total mercury load was found to be over 95% in aimara flesh. The content in several other metals of the control and the three fish-containing regimens has been assessed, along with that of the lyophilized aimara flesh (Table 2). Metals have been assayed by ICP-MS (Antellis, Toulouse, France). The diets and fish flesh levels were below the detection threshold for Ag (< 0.02 mg.kg^-1^), As (< 0.1 mg.kg^-1^), Au (< 0.05 mg.kg^-1^), Bi (< 0.02 mg.kg^-1^), Sb (< 0.5 mg.kg^-1^), Sn (< 0.5 mg.kg^-1^), Tl (< 0.05 mg.kg^-1^), and V (< 0.5 mg.kg^-1^). The RM1 control diet contained greater metal concentrations than aimara flesh probably due to the fact that plants accumulate heavy metals from soil. Nevertheless, aimara flesh in addition to mercury is also richer in selenium than RM1 diet, a known feature of fish flesh. Consequently, besides mercury, the low and mid level diets are not distinguishable from the control diet in terms of metal content, and the 7.5% fish diet contains twice as much selenium than the control diet.

**Table 1:** The composition of the diets used.^a^

		Diet (percentage of fish flesh in food)		
		
	Control diet	0.1%	1%	7.5%
				
Moisture	10	10	10	10
Fat	2.71	2.71	2.70	2.64
Protein	14.38	14.46	15.14	20.04
Fibre	4.65	4.65	4.61	4.35
Ash	6.00	6.00	5.99	5.90
**Carbohydrates**				
Starch	44.97	44.97	44.52	41.60
Sugar	4.05	4.05	4.01	3.75
Pectin	1.52	1.52	1.50	1.41
Hemicellulose	10.17	10.17	10.07	9.41
Cellulose	4.32	4.32	4.28	4.00
Lignin	1.68	1.68	1.66	1.55
**Fatty acids**				
Saturated fatty acids				
C12:0 Lauric	0.02	0.02	0.02	0.02
C14:0 Myristic	0.14	0.14	0.14	0.13
C16:0 Palmitic	0.31	0.31	0.31	0.31
C18:0 Stearic	0.04	0.04	0.04	0.05
Monounsaturated fatty acids				
C14:1 (w5) Myristoleic	0.02	0.02	0.02	0.02
C16:1 (w7) Palmitoleic	0.09	0.09	0.09	0.09
C18:1 (w9) Oleic	0.77	0.77	0.76	0.73
Polyunsaturated fatty acids				
C18:2 (w6) Linoleic	0.69	0.69	0.68	0.64
C18:3 (w3) Linolenic	0.06	0.06	0.06	0.05
C20:4 (w6) Arachidonic	0.13	0.13	0.13	0.13
C22:6 (w3) Cervonic (DHA)	< 0.01	< 0.01	< 0.01	0.02

^a ^Nutrients and compounds are given as their percentages in the diets.

**Table 2:** The metal composition of the diets used. ^a^

			Diet (percentage of fish flesh in food)		
			
Element	Aimara flesh	Control diet	0.1%	1%	7.5%
					
Al	< 2	41.1	41.1	40.7	38
Cd	< 0.02	0.064	0.064	0.064	0.059
Co	< 0.1	0.80	0.80	0.79	0.74
Cr	< 0.25	0.72	0.72	0.72	0.67
Cu	0.78	7.99	7.99	7.92	7.45
Hg (total)	5	0.001	0.005	0.062	0.520
Ni	< 0.25	0.39	0.39	0.39	0.36
Pb	0.091	0.165	0.165	0.164	0.159
Se	3.85	0.30	0.30	0.33	0.57
Zn	19.7	41.1	41.1	40.9	39.5

^a ^Metals are given in mg.kg^-1^.

### Mice treatment and tissue sampling

Subjects were naïve male mice of the C57Bl/6 Jico inbred strain obtained from IFFA Credo (Lyon, France) at the age of 3 weeks weighing 8.2 ± 0.1 g. They were socially housed in standard conditions: room temperature (23°C), 12/12 light cycles and *ad libitum *food and water. Experiments were performed in compliance with the European Community Council directive of 24 November 1986 (8616091 EEC). Four groups of 8 mice were fed for one month as follows: one with the control RM1 diet, and the three other ones with 0.1, 1, and 7.5% fish flesh supplemented RM1 diets. At the end of the exposure period, mice were subjected to a cross maze test, in order to quantify anxiety. Thereafter, mice were killed by decapitation, blood was immediately collected, and all the tissues were dissected for mercury quantification and gene expression analysis. For muscle fiber bioenergetics, gastrocnemius, a fast-twitch skeletal muscle was dissected and immediately placed in a cooled solution of buffer A containing 2.8 mM CaK_2_EGTA, 7.2 mM K_2_EGTA, 6.5 mM MgCl_2_, 5.7 mM Na_2_ATP, 15 mM phosphocreatine, 0.5 mM dithiothreitol, 50 mM potassium methanesulfonate, 20 mM imidazole, and 20 mM taurine (pH 7.1).

### Anxiety test using a cross maze

This test is one of the most widely used tests for assessing anxiety states of individuals [14,15]. The cross maze was elevated to 50 cm above the floor and consisted of two open and two closed arms (fenced on three sides). In such a maze, mice experience open bright spaces as worrying and closed dark ones as reassuring. Thus, open arms of the maze and especially their extremities will be experienced by the animals as deeply anxiety-producing places, centre as mildly anxiety-producing whereas closed arms will be felt as comforting places. Individuals were tested in the maze for 5 min as previously described [16]. Animals were placed in the centre of the maze with the nose pointing toward a closed arm; measures reflecting the anxiety state were measured, as follows: the time spent in the open arms, centre, and closed arms of the maze (data presented as percentage ratios of the time respectively spent in these zones to the total test time), the number of excursions into the open and closed arms, also expressed as percentage ratios, the time spent at the extremity of open and closed arms expressed in seconds, the total number of entries and exits from arms mostly reflecting the general activity of the mice. The maze was thoroughly cleaned and dried with clean tissues after each individual had been tested. All experiments were performed under normal laboratory illumination (1 × 100 W white light) during light phase of the light-dark cycle.

Statistical analysis of anxiety state parameters was performed with a non-parametric Kruskall-Wallis analysis of variance method followed by a Mann-Whitney U test.

### Mercury quantification

Total Hg concentrations in mice tissues were determined by flameless atomic absorption spectrometry. Analyses were carried out automatically after thermal decomposition at 750°C under an oxygen flow (AMA 254, Prague, Czech Republic). The detection limit was 0.01 ng Hg. The validity of the analytical methods was checked during each series of measurements using three standard biological reference materials (TORT2, DOLT2 and DOLT3); Hg values were consistently within the certified value range (data not shown). Stomach and intestines were washed from processed food and faecal matter before analysis.

### Gene expression analysis

Total RNAs were extracted from 40 mg of fresh hippocampus, liver, kidney, and muscle tissues using the Absolutely RNA Miniprep kit (Stratagene), according to the manufacturer's instructions. First-strand cDNA was synthesized from 5 μg total RNA using the Stratascript First-Strand DNA Synthesis kit (Stratagene). The cDNA mixture was stored at -20°C until its use in real-time PCR reaction. The accession numbers of the 9 genes used in our study are listed in Table 3. For each gene, specific primer pairs (see Table 3) were determined using the LightCycler probe design software (version 1.0, Roche). Real-time PCR reactions were performed in a LightCycler (Roche) according to the manufacturer's instructions: one cycle at 95°C for 10 min, and 50 amplification cycles at 95°C for 5s, 60°C for 5s and 72°C for 20s. Each 20 μl reaction contained 2 μl of reverse transcribed product template, 1 μl of master mix including the SyberGreen I fluorescent dye (Roche), allowing the monitoring of the PCR amplification, and the gene-specific primer pair at a final concentration of 300 nM for each primer. Reaction specificity was determined for each reaction from the dissociation curve of the PCR product. This dissociation curve was obtained by following the SyberGreen fluorescence level during a gradual heating of the PCR products from 60 to 95°C. Relative quantification of each gene expression level was normalized to the β-actin gene expression. The differential expression of a gene was calculated as the ratio of its expression, normalized to β-actin, in fish-contaminated condition to that in the control condition. Only the differential gene expression levels at least equal to or above 2 were considered.

**Table 3:** Specific primer pairs for the *Mus musculus *genes used. ^a^

Gene name	Accession number	Primer (5'-3')
		
β-*actin*	XR_004211	CACGGTGGGTAAGAGACAG^b^ AGGGGGAATGGTGAGCAG^c^
		
*Bax*	BC018228	AACTTCAACTGGGGCCG^b^ CACTGTCTGCCATGTGGG^c^
		
*CoxI*	NC_005089	TCACCCTAGATGACACATGAGC^b^ TGAAGCAAAGGCCTCTCAAAT^c^
		
*Fos*	NM_010234	CCGAAGGGAACGGAATAAGA^b^ GCAGGCAGGTCGGTGG^c^
		
*Gsta4*	NM_010357	AGACCACGGAGAGGCT^b^ CCTGACCACCTCAACATAGGG^c^
		
*Mt1*	NM_013602	ATGGACCCCAACTGCCTCCTG^b^ CAGCCCTGGGCACATTTGGAC^c^
		
*Mt2*	NM_008630	ATGGACCCCAACTGCCTCCTG^b^ CAGCCCTGGGAGCACTTCGCA^c^
		
*Sod2*	NM_013671	TCTCAACGCCACCGAGG^b^ AGACCCAAAGTCACGCT^c^
		
*Sod3*	NM_011435	TAGGACGACGAAGGGAGGT^b^ GGTCCCCGAACTCATGC^c^

^a ^Abbreviations: β-*actin*: cytoplasmic β-actin; *Bax*: Bcl2-associated X protein; *Cox*I: cytochrome *c *oxidase subunit I; *fos*: FBJ osteosarcoma oncogene; *Gsta4*: glutathione S-transferase, alpha 4; *Mt1*: metallothionein isoform 1; *Mt2*: metallothionein isoform 2; Sod2: mitochondrial superoxide dismutase; *Sod3*: extracellular superoxide dismutase.

^b^:upstream primer; ^c^:reverse primer.

Interindividual variability for each experimental condition was defined by mean ± standard deviation (*n *= 3). Significant differential gene expression levels between control mice and fish-fed mice in the four organs were determined using the nonparametric Mann-Whitney U-test (*p *< 0.05).

### Mitochondrial respiration measurements on skinned muscle fibers

Gastrocnemius and quadriceps muscular fibers (between 10 and 20 mg) collected on the posterior limbs of mice were permeabilized with saponin, a natural smooth detergent, in order to make mitochondria accessible to respiratory substrates added in the media. Bundles of fibers were incubated for 20 min in 5 ml of solution A containing saponin 50 μg/ml as described [17]. The bundles were then washed twice for 15 min in solution B (EGTA 10 mM, Mg2+ 3 mM, taurine 20 mM, dithiotreitol 0.5 mM, imidazole 20 mM, K+MES 0.1 M pH 7.0, phosphate 3 mM and 5 mg/ml fatty-acid-free bovine serum albumin) to remove saponin. All procedures were carried out at 4°C with extensive stirring. Finally, the preparations remained stable in the ice-cold solution B for 3 h. Mitochondrial oxygen consumption was monitored at 30°C in a 1 ml thermostatically controlled chamber equipped with a Clark oxygen electrode connected to a computer that gives an on-line display of the respiratory rate value (Hansatech, OXY1 system). The oxygraph cuvette contained one bundle of permeabilized muscles (around 12 mg) in 1 ml of solution B with Ap5A (di(Adenosine-5') pentaphosphate) 50 μM, iodoacetate 10 mM, EDTA 0.2 mM and the respiratory substrates (pyruvate 10 mM in the presence of malate 10 mM). State 3 was obtained by addition of 2 mM ADP. After each respiration measurement, the bundle of fibers was removed from the cuvette, dried and weighed to allow expression of the respiratory rates in ng atom O/min/mg of fibers. The respiratory control ratio (RCR) is defined as the ratio of state 3 (in the presence of ADP) to state 4 (in absence of ADP) respiratory rates.

### Cytochrome c oxidase activity

Cytochrome *c *oxidase activity was monitored by inhibiting the upstream components of the respiratory chain with rotenone and antimycin, and by using 3 mM ascorbate and 0.5 mM TMPD as an electron donor system. The respiratory rate was monitored using the polarographic method described above [17].

## Results

### Mercury bioaccumulation within mice organs

After 34 days of feeding with mercury-contaminated fish flesh, no differences in mass body weight were observed among the four groups of mice. They weighed 24.6 ± 0.7, 24.4 ± 1.5, 25 ± 1.3, and 24.8 ± 1.4 g for the control mice and the 0.1, 1, and 7.5% fish-fed mice, respectively. At the lowest fish dose (0.1%), hairs accumulated a mercury quantity 80-times above that of hairs in control mice (Table 4). Kidneys were the most mercury-impregnated organs, and for the 0.1 and 1% fish-containing regimens, contained a mercury concentration equivalent to that of hairs. At the lowest fish dose, the organs accumulating the highest mercury concentrations were, in decreasing order, kidneys (116 ng/g), muscles (8 ng/g), liver (6,7 ng/g), and brain structures (5 ng/g). At the highest fish dose (7.5%), mice hairs contained 10 ± 3 μg Hg/g, i.e. as much as in the hair of 84% of Wayanas Amerindians. In all the organs a gradation in mercury concentration was observed that was proportional to the fish flesh content and therefore to the intensity of the contamination pressure.

**Table 4:** Mercury bioaccumulation in various tissues. ^a^

Tissue (*n *= 3)	Control	Diet (Hg dose in food expressed in ng/g)		
		
		5.4	62	520
				
Hairs	1 ± 0.8	81 ± 47	1579 ± 165	10364 ± 3103
Kidneys	0.3 ± 0.1	116 ± 20	1435 ± 112	7730 ± 59
Liver	0.2 ± 0.04	6.7 ± 1.2	150 ± 14	1135 ± 324
Muscles	0.2 ± 0.2	8 ± 1	88 ± 2	543 ± 65
Hippocampus	NQ	5.9 ± 1	64 ± 5	417 ± 39
Brain	NQ	5 ± 1	63 ± 3	299 ± 31
Lung	NQ	2.5 ± 1.5	77 ± 18	1111 ± 232
Intestines	NQ	2.1 ± 0.9	70 ± 4	614 ± 90
Heart	NQ	1.8 ± 0.7	64 ± 12	576 ± 69
Spleen	NQ	1.5 ± 0.3	54 ± 4	517 ± 137
Stomach	NQ	1.2 ± 0.3	45 ± 7	317 ± 119
Skin	NQ	1.2 ± 0.4	15.3 ± 3	201 ± 28
Blood	NQ	1.1 ± 0.6	34 ± 4	298 ± 36

^a ^After one month feeding with fish-containing diets mercury was quantified in tissues (ng Hg/g fresh weight, mean ± SE). NQ: not quantifiable; below the threshold value.

### Impact of fish-containing diets on gene expression

Surveyed genes were chosen among those likely to have their expression modified by the mercury contamination. *Gsta4*, glutathione-S-transferase isoform 4, *Sod2*, mitochondrial superoxyde dismutase (SOD), and *Sod3*, extracellular SOD, are sentinels of an oxidative stress. *Cox*I, cytochrome *c *oxydase subunit 1, is a marker of the healthy status of the mitochondrial respiration, and *Mt1 *and *Mt2*, metallothionein isoforms 1 and 2, signal divalent metal intoxication. *Fos *and *Bax *can give clues about the general cellular stress and the proapoptotic cell commitment. Gene expressions were determined in hippocampus, skeletal muscles, liver, and kidneys, because these organs were those accumulating the highest mercury concentrations for the lowest fish regimen. When taking *β-actin *as the reference gene, basal gene expression showed wide variations for each gene among the four organs tested. The highest basal gene expression levels were seen in muscles for *Cox*I, *Sod2*, *Bax *and *Fos*, in kidneys for *Sod3*, in liver for *Gsta4*, and in hippocampus for *Mt1 *and *Mt2 *(Table 5). At the lowest tested fish regimen (0.1%), a differential expression was observed for *Cox*I and *Mt2 *genes in the liver, and for *Fos *in kidneys (Table 6). However, none of the tested genes responded in hippocampus and muscles. In liver, the mid-level diet induced *Cox*I, *Gsta4*, *Sod2*, *Sod3*, and *Mt2 *differential gene expression probably indicating an oxidative stress and a mitochondrial impact, i.e. the mitochondrial C*ox*I gene was stimulated 16-fold. However, the highest fish regimen did not further increase the differential gene expression pattern. In kidneys, *Bax*, *Cox*I, *Sod3*, and *Fos *genes responded to the mid-level diet (1%; 62 ng Hg/g), but went back to their basal expression levels for the highest fish regimen (7.5%; 520 ng Hg/g). Therefore, there is no simple relationship between contamination pressure and gene expression response in kidneys and liver. Only in hippocampus could we observe a relationship between diet mercury content and *Sod2*, *Mt1*, *Mt2*, *Bax *and *Fos *gene differential expressions.

**Table 5:** Comparative basal gene expressions in various tissues. ^a^

		Tissues			
		
Function	Gene	Hippocampus	Liver	Kidney	Muscles
					
Mitochondrial metabolism	*CoxI*	65.10^3 ^± 11.10^3^	2048 ± 1354	8192 ± 1214	104.10^4 ^± 84.10^4^
Oxidative stress	*Sod2*	8 ± 6.8	256 ± 104	128 ± 8.5	2048 ± 279
	*Sod3*	4 ± 1.9	2 ± 1.4	16 ± 2.2	8 ± 4.3
Detoxication process	*Mt1*	8 ± 2.5	4 ± 1.3	4 ± 0.9	4 ± 3.8
	*Mt2*	1024 ± 430	64 ± 3	64 ± 33	512 ± 482
	*Gsta4*	32 ± 27	512 ± 4.9	32 ± 4.4	256 ± 51
Apoptosis	*Bax*	8 ± 3.2	16 ± 2.1	16 ± 8.8	512 ± 189
	*Fos*	4 ± 3.7	0.25 ± 0.01	0.5 ± 0.4	16 ± 9.5

^a ^Gene expressions in brain, liver, kidneys, and skeletal muscles from control mice after one month feeding with vegetal diet (mean ± SE, *n *= 3). B-actin was the reference gene.

*Bax*: Bcl2-associated X protein; *Cox*I: cytochrome *c *oxidase subunit I; *fos*: FBJ osteosarcoma oncogene; *Gsta4*: glutathione S-transferase, alpha 4; *Mt1*: metallothionein isoform 1; *Mt2*: metallothionein isoform 2; *Sod2*: mitochondrial superoxide dismutase; *Sod3*: extracellular superoxide dismutase.

**Table 6:** Differential gene expressions in various tissues. ^a^

	Hippocampus			Liver			Kidneys			Muscles	
				
Food (ng Hg/g)	5.4	62	520	5.4	62	520	5.4	62	520	5.4	62
											
*CoxI*	=	=	=	**4**	**16**	**16**	=	**32**	=	=	=
*Sod2*	=	=	**16**	=	**2**	**2**	=	=	1/4	=	=
*Sod3*	=	**2**	=	=	**4**	**4**	=	**8**	=	=	**4**
*Mt1*	=	=	**8**	=	=	=	=	=	=	=	=
*Mt2*	=	**2**	**4**	**4**	**4**	**4**	=	=	=	=	=
*Gsta4*	=	=	=	=	**2**	**2**	=	=	=	=	=
*Bax*	=	=	**16**	=	=	=	=	**16**	=	=	=
*Fos*	=	**2**	**4**	=	=	=	**2**	**2**	=	=	=

^a ^Differential gene expressions after 1 month feeding with fish-containing diets. Three independent determinations per sample were carried out. The numbers are indicating the significant differential gene expressions. =: identical to control.

*Bax*: Bcl2-associated X protein; *Cox*I: cytochrome *c *oxidase subunit I; *fos*: FBJ osteosarcoma oncogene; *Gsta4*: glutathione S-transferase, alpha 4; *Mt1*: metallothionein isoform 1; *Mt2*: metallothionein isoform 2; *Sod2*: mitochondrial superoxide dismutase; *Sod3*: extracellular superoxide dismutase.

In summary, a net genetic response could be observed for mercury concentrations accumulated within tissues as weak as 0.15 ppm in the liver, 1.4 ppm in the kidneys, and 0.4 ppm in the hippocampus.

### Impact of fish-containing diets on muscle mitochondrial respiration

Whereas no significant influence of the fish-containing diets was observed at state 4, the respiration at state 3 was decreased dropping from 2.9 ± 0.7 for the control group to 1.6 ± 0.3 and 1.8 ± 0.4 ng atom O/min/mg fiber for the lowest and mid-level fish-containing diets, respectively (Table 7). The respiratory control ratio (RCR) was calculated for each bundle of muscle fiber from the ratio of the respiration at state 3 over that at state 4. RCR is indicative of the stimulatory effect of ADP on mitochondrial respiration since the ATP synthase is consuming both ADP and the transmembrane proton gradient generated by the respiration. In agreement with the observed decrease in state 3 respiration, RCR were significantly decreased as compared to the control group, with a 50, 34, and 42% loss for mice fed with 5.4, 62, and 520 ng Hg/g, respectively (Table 7). Cytochrome *c *oxidase activity was significantly decreased from 4.2 ± 0.8 ng atom O/min/mg fiber for the control group to 1.9 ± 0.27 natom O/min/mg fiber for the lowest fish-containing regimen (Table 7).

**Table 7:** Respiratory rates assayed on skinned muscle fibers. ^a^

		Diet (Hg dose in food expressed in ng/g)		
		
Oxygen consumption (ng atom O/min/mg fw)	Control diet	5.4	62	520
				
State 4 of respiration	1.6 ± 0.5	1.4 ± 0.2	1.3 ± 0.2	1.8 ± 0.4
State 3 of respiration	2.9 ± 0.7	*1.6 ± 0.3	*1.8 ± 0.4	2.1 ± 0.5
RCR	2.0 ± 0.2	*1.1 ± 0.2	*1.3 ± 0.2	*1.17 ± 0.05
COX activity	4.2 ± 0.8	*1.9 ± 0.3	3.2 ± 0.5	3.3 ± 0.3

^a ^Results are expressed as the mean ± SE, *n *= 4. Significant differences are indicated by an asterisk (*p *< 0.05).

### Impact of fish-containing diets on anxiety level

The cross maze study showed that mice fed 1 month with diets containing 0.1% or 1% of fish-flesh developed higher anxiety state behaviors compared to mice fed with control diet (Table 8). Anxiety was characterized by a general avoidance of open arms: decrease in the number of entries (*p *= 0.01 and 0.05), decrease of time spent in the open arms (*p *= 0.05 and 0.01), and at their extremities (*p *= 0.04 and 0.05). Parallel to this avoidance, a proclivity to stay in closed arms was developed by these mice: increase in the number of entries (*p *= 0.01 and 0.05), increase of time spent in the whole closed arms (*p *= 0.01 and 0.01), and at their extremities (*p *= 0.01 and 0.01). Noteworthy they also spent less time in the centre of the maze (*p *= 0.04 and 0.02), avoidance for this mildly anxiety producing place revealing a high level of anxiety in mice fed with 0.1 and 1% fish diets.

**Table 8:** Behavior of mice fed with mercury-containing diets in the cross maze test. ^a^

		Diet (Hg dose in food expressed in ng/g)		
		
Behavioral measures	Control diet (*n *= 6)	5.4 (*n *= 8)	62 (*n *= 8)	520 (*n *= 8)
				
Number of entries into open arms, %	28 ± 3.5	**13.7 ± 4.2	*21 ± 2.6	26.3 ± 3
Time spent in open arms, %	20.4 ± 2.9	*7.7 ± 2.9	*8.7 ± 1.6	14.7 ± 3.8
Time spent at the extremity of open arms, (sec)	30.9 ± 8.7	*13.4 ± 6.8	*14.6 ± 3.4	23.6 ± 8.7
Number of entries into closed arms, %	71.5 ± 4.2	**86.3 ± 4.2	*79 ± 2.6	73.7 ± 3
Time spent in closed arms, %	41.4 ± 2.8	**61.5 ± 4.3	**61 ± 2.6	49.4 ± 3.9
Time spent at the extremity of closed arms, (sec)	112.4 ± 13.6	**159.1 ± 14.2	**158.3 ± 8.5	116.5 ± 11.2
Time spent in center, %	38.3 ± 2	*31 ± 2.2	**30.2 ± 1.7	36.3 ± 1.7

^a ^Results are expressed as the mean ± SE. Significant differences are indicated by an asterisk (*p *< 0.05), or two (p < 0.01).

Surprisingly, in view of the results obtained with lighter diets, mice fed with the 7.5% fish-containing diet did not exhibit any statistically relevant differences in anxiety-like state behavior compared to controls.

## Discussion

Altogether, our results show that a vegetarian diet containing as little as 0.1% of mercury-contaminated fish is able to trigger, after only one month of exposure, bioenergetical disorders in skeletal muscles, a genetic response in liver and kidneys, and an increase in the anxiety-driven behavior of mice demonstrating that the aimara flesh is harmful. Although we cannot rule out that one or several toxic compounds, other than mercury, are present in the aimara flesh and act additionally to or synergistically with methylmercury, we now have solid arguments to incriminate methylmercury as the toxic compound being delivered by the fish flesh-containing diets to mice.

First, methylmercury is the only known toxic compound contaminating the food web of the Sinnamary River, and apart from clandestine gold mining activities, no sources of organic xenobiotics have been recorded so far in this part of the Amazonian jungle.

Second, the mercury accumulation in mice tissues is dependent on the diet fish content.

Third, gene expression studies are now powerful enough to discriminate and classify toxicants on the basis of unique gene expression profiles induced by putative toxic actions. Recently, this concept has been applied using DNA microarrays to evaluate the putative toxicity of environmental pollutants, yielding some chemical-specific gene expression patterns in mice tissues and cultured cells [18-21]. This concept also applies to metal intoxication: human lung cells have been exposed to cadmium chloride, sodium dichromate, nickel subsulfide or sodium arsenite. Using a 1200-gene microarray, it was shown that only three to seven genes overlapped among any two metal treatments [22]. In our hands, using a panel of selected genes, we could make a comparison of zebrafish genes differentially expressed in direct cadmium and trophic methylmercury contamination conditions. *p53 *and *sod1 *genes were specific to methylmercury whereas *hsp70*, *mt1*, and *pyc *were specific to cadmium. Among other genes, *bax*, *coxI*, *sod2*, and *mt2 *were common to both toxicants [23,24]. Worth noting, the same set of genes as those activated by methylmercury in zebrafish was also responding in mice fed with fish-containing diets. Indeed, this treatment stimulated the increased expression of *Cox*I, *Gsta4*, *Mt2*, *Sod2*, and *Sod3 *genes in liver, *Cox*I, *Bax*, *Fos*, and *Sod3 *genes in kidneys, and that of *Bax*, *Fos*, *Mt1*, *Mt2*, *Sod2*, and *Sod3 *genes in hippocampus. This pattern of gene expression was an expected response in the case of a mercurial contamination, and is unlikely to be caused by other toxic compounds. *Mt *gene overexpression is a hallmark of divalent cadmium and mercury exposure and has been observed in several animal tissues and cultured cells in addition to our zebrafish study: for instance in lungs of rats having inhaled mercury vapor [25], and in human hepatoma cells treated with cadmium or mercuric chloride [20]. In contrast, DNA microarray analysis showed that: 1/whereas cadmium chloride indeed triggered overexpression of *Mt1 *and *Mt2 *genes in mice liver, benzopyrene and trichloroethylene were unable to do so whatever the tested doses [18]; 2/whereas cadmium chloride and mercury chloride up-regulated *MT1 *gene in human hepatoma HepG2 cells, 2,3-dimethoxy-1,4-naphtoquinone exerted no effects, and phenol and *N*-nitrosodimethylamine down-regulated this gene [20]; 3/in rat liver, phenobarbital, gemfibrozil and clofibrate could not induce up-regulation of any of the genes we found stimulated in mice liver, with the exception of *Gst *gene in the case of phenobarbital. In fact, gemfibrozil and clofibrate rather down-regulated *Mt1 *and *Mt2 *genes [19]; 4/the same holds true in human hepatoma cells exposed to 2,3,7,8-tetrachlorodibenzo-*p*-dioxin, in which the only gene differentially expressed among those up-regulated in our mice liver was *Cox*I. However, it was 2.4-times down-regulated in this cell type [21]. Consistent with our findings, rats fed with mercury-contaminated rice produced near the Wanshan mercury mine in China were subjected to an oxidative stress materialized by an 87% increase in free radicals, a modification of the activity of superoxide dismutase, and the differential up-regulation of *Fos *gene in the hippocampus and cortex [26,27].

Fourth, our results on muscle mitochondrial respiration are fully concordant with the long-known effects of methylmercury on mitochondria of human and rat liver, i.e. state 3 respiration was inhibited by 10 to 50 nmol of methylmercury per mg of mitochondrial protein, and the resulting loss in membrane potential was the major cause of uncoupling [28]. In addition, purified beef heart cytochrome *c *oxidase is up to 50% inactivated by mercury chloride and ethylmercury [29], and in rats given methylmercury orally at a concentration of 5 μg/g per day for 12 days, mitochondria of skeletal muscles were affected by a decrease in cytochrome *c *oxidase and succinate dehydrogenase activities [30]. Methylmercury treatment also resulted in impaired mitochondrial dehydrogenase activity in cultured rat cerebellar granule cells [31] and mouse cerebellar neurons and astrocytes [32]. This is in keeping with our study on zebrafish fed with methylmercury-contaminated diet. After 49 days of contamination, state 3 mitochondrial respiration was reduced by 80%, and the cytochrome *c *oxidase activity reduced by 60% in saponin-permeabilized muscle fibers [33].

The biggest impact of fish diets on behavior, mitochondrial respiratory rates and kidney genes expression was observed with the low and mid level diets, not with the high level one. Although surprising at first glance, many experimental results are now showing that above a given dose of contaminant, the effects observed at low dose vanish or differ qualitatively. For instance, the effects of arsenic at 5 or 50 μM on human lung cells exposed for 4 hours were compared. Increasing the dose of arsenic from 5 to 50 μM did not simply increase the magnitude of the change in the same set of genes or induce additional genes response. Rather, a completely different pattern of gene response between the lower and the higher dose was observed. Over the 1200 genes examined at both doses, only 16 of the 160 affected genes were altered at both doses [34]. In another study authors carried out a serial analysis of gene expression (SAGE) in kidneys from mice exposed to chronic or acute uranyl nitrate contamination [35,36]. Only 16 genes were common to both SAGE lists and expressed the same way; 147 genes were differentially regulated in either one of the two conditions; 10 genes were common to both SAGE lists but expressed the opposite way, i.e. up-regulated under chronic exposure but down-regulated under acute exposure. These comparative patterns of gene expression data indicate that when shifting from chronic to acute exposure the intensity of gene response does not increase as might have been expected but rather that the qualitative nature of the gene response is completely changed resulting in a modified tissue metabolism. In keeping with this, it has been shown that: a/uranium is an endocrine disrupter in mice at low but not at high doses [37]; b/after 7 days of exposure, copper induced in the aquatic plant *Hydrilla verticillata *an increase of superoxide dismutase, glutathione peroxidase and catalase activities at low but not at high doses [38]; c/cadmium triggered greater genotoxic damages on *Xenopus laevis *larvae at 0.5 than at 1 mg/l [39]; d/carbon nanotubes induced in rainbow trout gills and intestine an increase of the Na^+^K^+^-ATPase activity at low but not at high doses [40]; e/nanofullerenes induced in largemouth bass gills and liver a change in the pattern of lipid peroxidation at 0.5 ppm but not at 1 ppm [41]. These results suggest a model in which low doses of pollutant cause mild effects compatible with life, allowing animal resistance-through adaptive response, whereas higher doses trigger acute effects threatening animal's life, resulting in general stress instead of adaptive response. Additionally, the highest regimen is rich in fish flesh and it has been argued that the beneficial influence of nutrients from fish may counter any adverse effects of MeHg on the developing nervous system [42].

When addressing the question as to whether mouse is a pertinent model for the mercurial intoxication of the Wayana Amerindians, a good criterion consists in a comparison of the mercury concentrations impairing cell life, i.e. resulting in cell death or limited cell viability, among various cell lines from different origins (Table 9) including bacteria [45], yeast [46], clam [47], worm [48], mosquito [49], chicken [50], fish [51], rabbit [52], rat [31,53,54], mouse [32,53,55,56], and man [57-59]. Remarkably, most of these values are ranging between 1 to 10 μM methylmercury whatever the considered organism, from bacteria to man, and for different cell types. Therefore, mercury toxicity does not depend on the species, the phylum, the global organism's metabolism, the body weight, or the organism's life span. Once inside a cell, a bolus of mercury will display its toxicity whatever the organism harboring that cell. Another pertinent argument making mice a good model lies in the tissue concentrations for which a genetic response was observed. In hippocampus 5 genes over 8 tested were up-regulated for a total mercury tissue concentration of 417 ng/g (in the case of the 7.5% diet), corresponding to brain mercury levels intermediate between reported acute and chronic exposure Minamata cases. In this brain structure, 3 genes over 8 tested were up-regulated for a total mercury tissue concentration of 64 ng/g (in the case of the 1% diet). This latter value is in the range of the mercury concentrations found in the brains of chronically exposed patients in the Minamata region 5 years after the Chisso company ceased to pollute the Minamata bay [43]. As a mean of comparison, the median total mercury concentration in the occipital cortex of human Norwegian individuals (*n *= 30) without occupational exposure to mercury was found to be 12 ng/g [12]. The reported 90-percentile value, 28 ng/g, is just 2.3-times below the mice hippocampal mercury concentration resulting in the up-regulation of *fos*, *mt2*, and *sod3 *genes (table 6). Therefore, after just one month of feeding with a diet containing 1% aimara flesh, mice hippocampus mercury level was equivalent to that of human brains from heavy fish consumers. In mice kidneys, 4 genes over 8 tested responded to a total mercury tissue concentration of 1.4 μg/g (in the case of the 1% diet). The total mercury mean concentrations in human kidneys are 0.7 μg/g for women (*n *= 22) and 0.4 μg/g for men (*n *= 17) with an overall distribution range of 0.04–2.1 μg/g. Only one person over 39 experienced a mercury occupational exposure. The fish consumption habit of 27 out of these 39 persons could be recorded: 6 were consuming less than 1 fish meal per week, 16 were consuming 1 fish meal per week, and 2 more than 1 fish meal per week [11]. Therefore, a net genetic response was observed in kidneys from mice fed 1% aimara flesh over one month, when tissue mercury concentration was equivalent to the highest values found in human kidneys from modest fish consumers. The same regimen yielded a blood total mercury concentration of 34 ± 4 μg/L, comparing easily with the blood mean mercury level of 54 ± 35 μg/L found in humans inhabiting Amazonian villages along the Tapajós River and eating a mean of 8.2 ± 4.9 fish meal per week [44].

**Table 9:** Toxic effects of mercury on various cell lines.

Organisms and species	Cell types	Mercury effects	Ref.
			
Bacteria *Escherichia coli*	Strains devoid of R plasmids	Growth inhibition: MIC = 11.5 μM HgCl_2_.	[45]
Yeast *Saccharomyces cerevisiae*	Strain W303B	Growth inhibition: MIC = 2 μM MeHgCl at 24 h.	[46]
Clam *Mya arenaria*	Hemocytes	Phagocytosis inhibition: IC_50 _= 0.44 μM MeHgCl at 18 h.	[47]
Earthworm *Lumbricus terrestris*	Coelomocytes	Phagocytosis inhibition: IC_50 _= 0.1 μM MeHgCl and 0.5 μM HgCl_2 _at 18 h.	[48]
Mosquito *Aedes albopictus*	Cell line C6/36	Cell viability: for serum deprived cultures, LD_50 _= 2.1 μM MeHgCl and 2.5 μM HgCl_2 _at 24 h; for cultures with fetal calf serum, LD_50 _= 5.5 μM MeHgCl and 12 μM HgCl_2 _at 24 h. Cell growth inhibition: IC_50 _= 1 μM MeHgCl and 18.4 μM HgCl_2 _at 18 days.	[49]
Chicken	Retinal cells	Cell viability: 49% cell death with 10 μM MeHgCl at 6 h.	[50]
Fish fathead minnow	CCL-42	Cell viability: EC_50 _= 1.55 μM MeHgOH at 96 h.	[51]
Rabbit	Renal proximal tubule cells	Cell viability: LC_50 _= 6.1 μM MeHgCl and 34.2 μM HgCl_2 _at 24 h.	[52]
Rat	Cerebellar granule cells	50% apoptotic cells with 1 μM MeHgCl for 9 h; 30% apoptotic cells and 60% reduction in mitochondrial dehydrogenases with 2.5 μM MeHgCl for 1 h.	[31]
Rat	Embryonic neural stem cells	90% cell death with 0.5 μM MeHgCl for 24 h; 37% apoptotic cells with 0.1 μM MeHgCl for 24 h.	[53]
Rat	Splenocytes Leukocytes	Cytolethality: 8 μM MeHgCl for 24 h.	[54]
Mouse	Cerebellar neurons	50% reduction in mitochondrial activity with 5 μM MeHgCl for 1 h.	[32]
	Cerebellar astrocytes	40% reduction in mitochondrial activity with 5 μM MeHgCl for 1 h.	
Mouse	Macrophages	Cell death: 20 μM MeHgCl for a few days.	[55]
Mouse	Peritoneal neutrophils	Necrotic cell death: 15 μM MeHgCl for 13 min.	[56]
Mouse	Multipotent neural stem cell line C17.2	45% cell death with 2 μM MeHgCl at 24 h; 20% apoptotic cells with 0.5 μM at 24 h.	[53]
Man	YAC-1 murine Moloney virus transformed lymphoma cell line	50% cell death with 25 μM MeHgCl at 4 h.	[57]
Man	T lymphocytes	Cell viability: 8 μM MeHgCl at 24 h.	[58]
	Monocytes	Cell viability: 8 μM MeHgCl at 4 h.	
Man	Neurons	Cell viability: LC_50 _= 6.5 μM MeHgCl at 24 h.	[59]
	Astrocytes	Cell viability: LC_50 _= 8.1 μM MeHgCl at 24 h.	
	Neuroblastoma cells	Cell viability: LC_50 _= 6.9 μM MeHgCl at 24 h.	

Ref.: references; EC_50 _: median effective concentration; HgCl_2 _: mercury chloride; IC_50 _: median inhibitory concentration; LC_50 _: median lethal concentration; LD_50 _: median lethal dose; MeHgCl: methylmercury chloride; MeHgOH: methylmercury hydroxide; MIC: minimal inhibitory concentration.

## Conclusion

The 7.5% fish-containing diet resulted in Hg brain concentrations equivalent to acute exposure cases and therefore cannot be useful to mimic human environmental cases. The 1% fish-containing diet yielded after only one month exposure, blood, kidney, and brain Hg concentrations in the range of what has been recorded in human blood, kidneys, and brains of heavy fish consumers in a general population. The 0.1% fish-containing diet brings to mice the same mercury contamination pressure as that afflicting the Wayana Amerindians assuming a Hg trophic transfer rate of 100%, and it can be expected that after several months, the mercury levels in mice tissues be equivalent to those observed after one month of feeding with diet containing 1% fish flesh. Since the 0.1% fish-containing regimen proved to affect gene expression, muscle mitochondrial respiration, and triggered an anxiety-driven behavior in mice, our study will be pursued with such a regimen for an extended time length encompassing the mouse lifespan in order to get a precise panorama of the impact of mercury-contaminated fish consumption all along the animals' life.

## Abbreviations

ADP: adenosine diphosphate; ATP: adenosine triphosphate; cDNA: complementary desoxyribonucleic acid; DNA: desoxyribonucleic acid; dw: dry weight; EDTA: ethylenediaminetetraacetic acid; EGTA: ethylene glycol tetraacetic acid; MES: morpholinoethanesulfonic acid; PCR: polymerase chain reaction; ppm: μg/g; RCR: respiratory control ratio; RNA: ribonucleic acid; SOD: superoxide dismutase; TMPD: tetramethyl phenylendiamine.

## Competing interests

The authors declare that they have no competing interests.

## Authors' contributions

JPB is supervising the program entitled "Effets du mercure sur la santé des ecosystèmes aquatiques et des populations humaines" ("Mercury impacts on aquatic ecosystems and human populations health") funded by the French National Research Agency. JPB conceived the study and participated in its design and coordination, participated in the mice tissue sampling, and wrote the manuscript. NB and GB performed the skinned muscle respiration studies. DB participated in the study design and in the mice tissue sampling. MF participated in the study design and aided in the preparation of the manuscript. PG participated in the study design, in the mice tissue sampling, and carried out the gene expression analysis. AM participated in the study design and in the mice tissue sampling, and was responsible for the behavioral study. RMB participated in the study design and in the mice tissue sampling design, and was responsible for the mercury quantitations. CM participated in the mice tissue sampling and provided expert skills in mice dissection. VP carried out the mercury quantitations. JNP carried out the behavioral assay. RR participated in the study design and in the mice tissue sampling, and was responsible for the muscle respiration study. WR participated in the study design and aided in the preparation of the manuscript. MS participated in the study design. ML analyzed the behavioral data, built the corresponding table, and contributed to the preparation of the manuscript. All the authors read and approved the final manuscript.
